# Improving communication between the general practitioner and the oncologist: a key role in coordinating care for patients suffering from cancer

**DOI:** 10.1186/s12885-020-06993-0

**Published:** 2020-06-01

**Authors:** Vladimir Druel, Laetitia Gimenez, Kim Paricaud, Jean-Pierre Delord, Pascale Grosclaude, Nathalie Boussier, Marie-Eve Rougé Bugat

**Affiliations:** 1 Department of Primary Care, University of Toulouse, 133 Route de Narbonne, 31062 Toulouse, France; 2 Oncology Unit, Auch Hospital, Auch, France; 3 Paul Sabatier University, Toulouse III, 133 Route de Narbonne, 31062 Toulouse, France; 4 Inserm U1027, Faculty of Medicine, 37 allées Jules Guesde, 31073 Toulouse, France; 5 Department of Internal Medicine, Toulouse University Hospital, 29 Rue Emile Lecrivain, 31077 Toulouse, France; 6 Institut Universitaire du Cancer de Toulouse-Oncopole, 1 Av. Irène Joliot-Curie, 31100 Toulouse, France; 7 Onco-occitanie, 1 Av. Irène Joliot-Curie, 31059 Toulouse, France

**Keywords:** Cancer, Communication, Disclosure, General practitioner, Oncologist

## Abstract

**Background:**

Patients suffering from cancers are increasingly numerous in general practice consultations. The General Practitioner (GP) should be at the heart of the management of patients. Several studies have examined the perceptions of GPs confronted with the patient suffering from cancer and the relationships of GPs with oncologists, but few studies have focused on the patients’ perspective. We studied the three-way relationship between the oncologist, the GP, and the patient, from the patient’s point of view.

**Methods:**

A questionnaire validated by a group consisting of GPs, oncologists, nurses, an epidemiologist and quality analyst, was administered over a three-week period to patients suffering from cancer receiving chemotherapy in a day hospital.

**Results:**

The analysis was based on 403 questionnaires. Patients had confidence in the GP’s knowledge of oncology in 88% of cases; 49% consulted their GP for pain, 15% for cancer-related advice, and 44% in emergencies. Perceived good GP/oncologist communication led patients to turn increasingly to their GP for cancer-related consultations (RR = 1.14; *p* = 0.01) and gave patients confidence in the GP’s ability to manage cancer-related problems (RR = 1.30; *p* < 0.01). Mention by the oncologist of the GP’s role increased the consultations for complications (RR = 1.82; *p* < 0.01) as well as recourse to the GP in an emergency (RR = 1.35; *p* < 0.01).

**Conclusion:**

Patients suffering from cancer considered that the GP was competent, but did not often consult their GP for cancer-related problems. There is a discrepancy between patients’ beliefs and their behaviour. When the oncologist spoke to patients of the GP’s role, patients had recourse to their GP more often. Systematically integrating a GP consultation to conclude cancer diagnosis disclosure, could improve management and care coordination.

## Background

The number of cancer diagnoses increased by 1.6 million worldwide between 2008 and 2012 [1] and by 0.7 million in Europe between 2006 and 2012 [2]. While the annual increase of cancer incidence in France is about 1% [3], mortality decreased between 1985 and 2012 [4]. More and more patients consulting their general practitioner (GP) have, or have had, cancer [5]. The GP has a central role in the care of patients with cancer [6]. The GP intervenes at all stages of care (prevention, screening, diagnosis, treatment, follow-up, education) [7–9]. For greater equity in the management of patients suffering from cancer, a French National Cancer Plan has been implemented [10] to standardize its management. The plan proposes that the GP should be at the center of the care of patients suffering from cancer.

Collaborative care is the process through which different professional groups work together to improve healthcare quality [11]. Providers working as a team promote improved communication, coordination of care, and patient-centred shared decision making [12, 13]. A clear definition of the roles of every participant [14] and a good relationship between the patient and their GP, together with good communication between the GP and the oncologist, make for optimal care [15].

There is a general lack of trust on both sides between oncologist and GP, and communication is poor [16]. GPs often feel alienated from the hospital system [15, 17] and demand that their role in the management of patients with cancer is reinforced. For their part, 67% of patients would like to see follow-up shared between the GP and the oncologist, that is, they would like the GP to deal with both cancer-related problems (in collaboration with the specialist) and also with unrelated problems in a holistic and patient-centred approach [18, 19]. The perspective of oncologists and GPs on this question of communication have previously been examined in the literature. There is a lack of communication [12, 13, 20] that requires the implementation of inter-professional collaboration and communication tools [20] to promote direct communication (telephone) with a patient-centred approach [12]. But few studies have focused on the patients’ perspective. Those interested in it show the importance of the role of the GP and a desire on the part of patients to see an increase in the role of the GP in oncology management [21–24]. The GP participates predominantly in supportive care. It is suggested that improved communication between oncologist and GP as perceived by the patient improves the involvement of the GP [24]. In this context, we are particularly interested in the management of a patient by an oncologist and GP from the point of view of the patient. We explored this three-way relationship with the perspective of improving the coordination of cancer care.

## Methods

In this purpose, a cross-sectional survey questionnaire was conducted in a sample of patients between the 15th of July and the 1st of August 2015. Patients coming for chemotherapy were invited to participate in the study and, if they consented, to complete a paper questionnaire in the day hospital of the Comprehensive Cancer Center of Toulouse (south-west France). The patients included in the study were patients with cancer over 18 years old, receiving chemotherapy in the active phase of treatment, including a patient with maintenance treatment. Patients with haematological disease or those who were not followed up by a GP were excluded. There was no other social or medical criterion of exclusion. Patients who did not accept to complete the questionnaire were not included. Questionnaires were distributed when the patients arrived for treatment and collected upon their departure. The same patient could answer the questionnaire only once. In order to limit desirability bias, the questionnaires were distributed and collected by the para-medical team (and not the medical team). These data were manually transcribed onto a spreadsheet before analysis.

This study was approved by the ethical committee of the university department of Toulouse (22nd of June 2015).

### Questionnaire

The questionnaire was developed and validated after two working meetings by three GPs, two oncologists, four nurses, two nursing assistants, an epidemiologist and a quality analyst at the cancer center. It was tested by three patient volunteers, which allowed some adjustments to be made. These adjustments concerned only the wording of the questions, in order to improve their understanding. No further changes were necessary. The questionnaire included four parts: (1) the patients’ characteristics, (2) their relationships with their GP, (3) GPs and cancer (history of the disease and GP involvement), and (4) relationships between GPs and oncologists from the patients’ perspective. The questionnaire was written in French, and consisted of 24 closed-ended questions and one open-ended question. The questionnaire used is available in a supplementary file (see Additional files 1 & 2).

### Statistical analyses

All the characteristics of the population at inclusion, as well as the variables of interest, are described in terms of numbers and percentages (and average for each age group). Following this descriptive analysis, a comparative analysis was carried out to investigate the factors that could be associated with the change of GP. Furthermore, we analysed the associations between the patient’s opinion of the relationship between GP and oncologist, and reasons to consult the GP, in parts (3) and (4) of the questionnaire. In this section, we focused on the patient’s perception of the interaction between GP and oncologist (in the cases when the oncologist mentioned the GP’s role, the GP and the oncologist communicated, and the GP’s opinion was taken into account). We analyzed whether the patient’s feelings influenced his or her own behaviour towards the management of oncology health problems, including complications of the oncological treatment, pain, oncological management, emergencies, as well as the competence of the GP in these situations. In order to compare the qualitative variables, a chi-squared test was performed. Each bivariate analysis considered only those patients who responded to the different items analysed and the reference variable is the first mentioned in the sentence. We calculated the Risk Ratio (RR) to improve the understanding of the analyses. Statistical tests were two-sided at the 5% level of significance. All analyses were done using STATA software version 8.

## Results

**Fig. 1:**
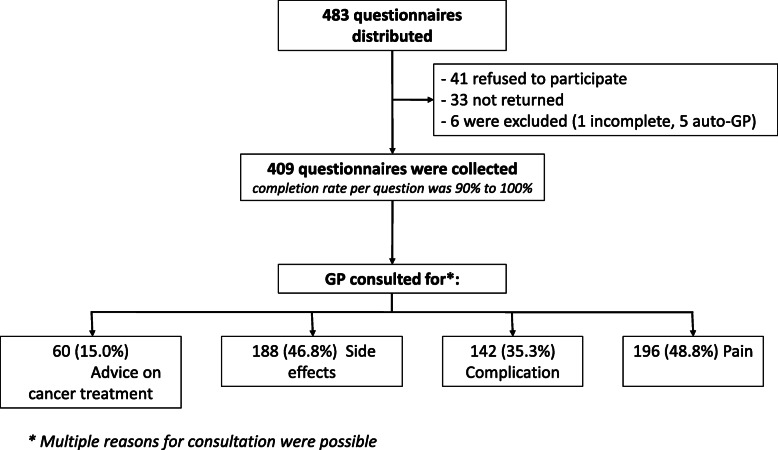
Flow chart of response rate

A total of 483 questionnaires were distributed, 41 patients declined to answer and 33 did not return the questionnaire, so 409 questionnaires were collected, and 403 analysed. Six were excluded: 5 because only the first page had been completed, and one because the respondent was his own GP. The response rate per question was 90 to 100% (Fig. 1).

Most patients were women (85.1%), with a median age of 60 (mean 59.8) (Table 1). One hundred ninety-seven patients had the same GP for at least 15 years and 60.7% (*n* = 244) had visited their GP in the month before the survey. Only 4.5% (*n* = 18) had not visited their GP for more than 6 months. About one in 5 patients had changed their GP since the cancer diagnosis. This change was not associated with the GP’s participation in diagnosis significantly even if there was a trend (RR = 0,67, *p* = 0,06) (Table 2).

**Table 1:** Characteristics of patients with cancer who responded to the questionnaire (*n* = 403)

Characteristics of the study population		n (%)
Sex	Men	60 (14.9%)
	Women	343 (85.1%)
Age (years)	< 50	74 (18.4%)
	50 to 75	287 (71.2%)
	> 75	42 (10.4%)
Time since diagnosis (years)	< 1	206 (51.2%)
	≥ 1	196 (48.8%)
**GP/ patient relationship**		
Duration of care by the same GP (years) *With change of GP since cancer diagnosis*	< 5	97 (24.1%)
	5 to 15	109 (27.0%)
	> 15	197 (48.9%)
		*71 (17.7%)*
Last visit to GP (month)	< 1	244 (60.7%)
	1 to 3	121 (30.1%)
	4 to 6	19 (4.7%)
	> 6	18 (4.5%)
	ND	1
Participation of GP in diagnosis	Yes	211 (52.8%)
	ND*	3
**GP and cancer**		
Last consultation was cancer-related	Yes	289 (71,7%)
	ND*	0
GP’s knowledge of cancer	Yes	353 (88.5%)
	ND*	4
GP is kept informed	Yes	336 (85.9%)
	ND*	12
GP knows about last treatment change	Yes	347 (86.1%)
	With no treatment change	131 (32.5%)
GP consulted for:		
Advice on cancer treatment	Yes	60 (15.0%)
	ND*	2
Side effects	Yes	188 (46.8%)
	ND*	1
Complication	Yes	142 (35.3%)
	ND*	1
Pain	Yes	196 (48.8%)
	ND*	1
Professional consulted in an emergency	GP	173 (43.8%)
	Emergency department	28 (7.1%)
	Specialized cancer center	194 (49.1%)
	ND*	8
GP is available in case of emergency	Yes	242 (62.5%)
	ND*	16
GP is able to manage their care	Yes	225 (58.3%)
	ND*	17
**GP/oncologist relationship**		
Communication between oncologist and GP	Yes	239 (66.2%)
	ND*	42
GP participates in decision-making	Yes	100 (27.3%)
	ND*	37
GP’s role mentioned by oncologist	Yes	164 (42,4%)
	ND*	16

*ND: not determined

**Table 2:** Factors associated with a change of general practitioner

		Change of general practitioner			
		Yes	No	RR	*p*-value**
		71 (17.7%)	331 (82.3%)		
GP participation in cancer diagnosis	No	40 (21.3%)	148 (78.7%)	1	0.06
	Yes	30 (14.2%)	181 (85.8%)	0.67	
	ND*	1	2		
Last visit to GP (month)	≤ 3	195 (53.9%)	167 (46.1%)	1	0.22
	> 3	16 (43.2%)	21 (56.8%)	1.25	

*ND: not determined ** Chi-squared test

### General practitioner/patient relationship

Half the patients stated that the GP had participated in the initial diagnosis (*n* = 211, 52.8%). Participation in diagnosis was not significantly associated with change in the frequency of GP visits (RR = 0.82, *p* = 0.74). Nine out of 10 patients considered that the GP was knowledgeable about the patient’s specific type of cancer (i.e., knew how to support their treatment and survivorship care). The reason for the most recent GP consultation was cancer-related in 71.7% of cases (*n* = 289). Multiple reasons for consultation were possible (Fig. 1).

According to the patients’ answers, 42.4% of the oncologists had mentioned the GP’s role; when the oncologist had involved the GP by mentioning the GP’s role in management, this had an impact on the proportion of patients who consulted their GP. Thereafter, they consulted the GP more often for the management of side effects (RR = 1.34, *p* ≤ 0.01), pain (RR = 1.55, *p* ≤ 0.01), cancer-related complications (RR = 1.82, *p* ≤ 0.01), and for specific advice on their cancer treatment (RR = 1.83, *p* ≤ 0.01) (Table 3). When the patient considered that the GP and the oncologist were in communication with each other, the proportion of patients consulting for cancer treatment advice increased.

**Table 3:** Association between patient’s opinion of relationship between GP and oncologist, and reasons for GP consulting

		The patient considers that:					
**The patient consults their GP for:**		The oncologist mentioned the GP’s role		The GP and the oncologist communicate		The GP’s opinion is taken into account	
		Yes	No	Yes	No	Yes	No
Side effects	Yes 188 (46.8%)	91 (55.5%)	92 (41.4%)	121 (50.6%)	56 (46.3%)	48 (48.0%)	124 (46.8%)
	No 214 (53.2%)	73 (44.5%)	130 (58.6%)	118 (49.4%)	65 (53.7%)	52 (52.0%)	141 (53.2%)
	**RR**	**1.34**	**1**	**1.09**	**1**	**1.03**	**1**
	*p*-value*	< 0.01		0.44		0.84	
Pain	Yes 196 (48.8%)	101 (61.6%)	88 (39.6%)	119 (49.8%)	60 (49.6%)	57 (57.0%)	126 (47.5%)
	No 206 (51.2%)	63 (38.4%)	134 (60.4%)	120 (50.2%)	61 (50.4%)	43 (43.0%)	139 (52.5%)
	**RR**	**1.55**	**1**	**1.00**	**1**	**1.20**	**1**
	*p*-value*	< 0.01		0.97		0.11	
Cancer-related complications	Yes 142 (35.3)	78 (47.6%)	58 (26.1%)	92 (38.5%)	41 (33.9%)	47 (47.0%)	81 (30.6%)
	No 260 (64.7)	86 (52.4%)	164 (73.9%)	147 (61.5%)	80 (66.1%)	53 (53.0%)	184 (69.4%)
	**RR**	**1.82**	**1**	**1.14**	**1**	**1.54**	**1**
	*p* – value*	< 0.01		0.39		< 0.01	
Advice on cancer treatment	Yes 60 (15.0)	34 (20.7%)	25 (11.3%)	47 (19.7%)	11 (9.2%)	29 (29.0%)	26 (9.8%)
	No 341 (85.0)	130 (79.3%)	196 (88.7%)	192 (80.3%)	109 (90.8%)	71 (71.0%)	238 (90.2%)
	**RR**	**1.83**	**1**	**2.14**	**1**	**2.94**	**1**
	*p*-value*	0.01		0.01		< 0.01	
Emergency	Yes 173 (43.8)	82 (51.25%)	83 (37.9%)	111 (47.2%)	51 (42.9%)	60 (61.2%)	98 (37.7%)
	No 222 (56.2)	78 (48.75%)	136 (62.1%)	124 (52.8%)	68 (57.1%)	38 (38.8%)	162 (62.3%)
	**RR**	**1.35**	**1**	**1.10**	**1**	**1.62**	**1**
	*p*-value*	< 0.01		0.43		< 0.01	
**The patient considers** **that the GP is:**							
Able to manage their care	Yes 225 (58.3%)	99 (64.3%)	118 (54.1%)	148 (64.3%)	59 (49.6%)	73 (77.7%)	132 (51.2%)
	No 161 (41.7%)	55 (35.7%)	100 (45.9%)	82 (35.7%)	60 (50.4%)	21 (22.3%)	126 (48.8%)
	**RR**	**1.19**	**1**	**1.30**	**1**	**1.52**	**1**
	*P* value*	0.05		< 0.01		< 0.01	
Available in case of emergency	Yes 242 (62.5%)	105 (67.7%)	130 (59.9%)	162 (69.8%)	62 (52.5%)	74 (77.9%)	147 (57.2%)
	No 145 (37.5%)	50 (32.2%)	87 (40.1%)	70 (30.2%)	56 (47.5%)	21 (22.1%)	110 (42.8%)
	**RR**	**1.13**	**1**	**1.33**	**1**	**1.36**	**1**
	*p*-value*	0.12		< 0.01		< 0.01	

RR = Relative risk * Chi-squared test

Turning to the GP in an emergency was also associated with mention of the GP’s role by the oncologist (RR = 1.35, *p* < 0.01) (Table 3). The GP was considered to be available in an emergency by 62.5% (*n* = 242) of patients, and competent to deliver emergency care by 58.3% (*n* = 225). In addition, there was a very strong association between confidence in the GP’s availability and in their ability to deliver emergency care (RR = 3.56, *p* < 0.01). Confidence in ability to deliver emergency care was associated with good GP/oncologist communication (RR = 1.33, *p* < 0.01) (Table 3).

### General practitioner/oncologist relationship

The majority of patients (*n* = 336, 85.9%) considered that the GP was kept informed by the oncologist and that the GP knew about the most recent change in their treatment (no treatment change *n* = 131) (Table 1). Two-thirds of patients considered that the GP and the oncologist communicated with each other (*n* = 239, 66.2%), and this belief was associated with consulting the GP about cancer treatment (RR = 2.14, *p* = 0.01), confidence in the GP’s availability (RR = 1.33, *p* < 0.01) and in their ability to manage the patient’s care (RR = 1.30, *p* < 0.01) (Table 3).

Nearly one quarter of patients (*n* = 100, 27.3%) considered that the GP’s opinion was taken into account in medical decision-making [Table 1). Those who considered that their GP was involved were more likely to turn to their GP in an emergency (RR = 1.62, *p* < 0.01), and this was also correlated with confidence in their availability (RR = 1.36, *p* < 0.01) and competence (RR = 1.52, *p* < 0.01) (Table 3).

## Discussion

According to the majority of patients (*n* = 353, 88.5%), the GP was knowledgeable and capable, but few patients (*n* = 60, 15.0%) consulted their GP for cancer-related problems. There was a discrepancy between patients’ beliefs and their behaviour. Mention by the oncologist of the GP’s role increased patients’ recourse to the GP. There was a correlation between patients consulting their GP in an emergency and their perception of the role attributed to the GP by the oncologist. The patient’s understanding of the GP’s role was determinant in the quality of the care he/she received. Good communication between the general practitioner and the oncologist improved the quality of coordination, affecting the patient’s life.

### Strengths and limitations

The first strength of our study is that patients’ data were collected in a day hospital treating all types of cancer, which, moreover, is the regional comprehensive center that recruits patients from the whole Midi-Pyrénées region in France. The day hospital treats a large number of patients (1249 chemotherapy sessions a month, or about 60 sessions a day) who have various types of cancer and are followed by different oncologists. The size of the center allowed us through this project to study a large population of patients, with different oncology and GP practices.

The importance given to the patient’s point of view allowed us to focus on patients’ feelings, needs and perceptions.

A large proportion of patients were women (*n* = 343, 85.1%) as there is a large proportion of women treated for breast cancer (61.4% referred by breast specialists and 9.8% by gynaecologists) in the day unit. Worldwide, breast cancer is the cancer with the highest incidence in women, with 1.7 million new cases in 2012 (or 25% of cancers in women) and 0.5 million deaths [2]. In addition, most treatment lines for breast cancer are administered in the day hospital, with a long survival that extends the duration of care. All in all, this leads to an over-representation of breast cancer and women in our study. So, these data are generalizable to patients suffering from breast cancer and may be reflective of the experience of patients with other cancers.

The main limitation of this study is that it analyses patient-reported data only with a high memory bias. In this paper, we wished to explore the relationships between different factors that might explain a patient’s reticence to consult their GP for their cancer care. Another shortcoming is that the study does not highlight the efforts made by the main medical practitioners (oncologist and GP) but rather focuses on the effect of the medical efforts on patients’ attitudes towards their GP, and the perception of the communication by the patient, which influences the patients’ confidence towards his/her various interlocutors. Another limitation of our study is a mono-centric design, which has a center effect and a selection bias. We chose a quantitative instead of a qualitative method to measure the lack of communication between oncologist and GP, and compared our results with the literature.

We wished to look for association between different factors that could explain patients’ reticence to consult their GP for their cancer care. We sought to have a large sample of patients with cancers in our study in order to be as representative as possible of our study population despite our mono-centric study. In addition, closed-ended questions allowed us to assess the presence of associations between patients’ responses. These elements justify our choice of a quantitative rather than a qualitative method.

### Comparison with existing literature

Shared decision-making needs to take place with the patient (patient-centered), because it is an important step in the patient’s life that impacts their perception of the disease [25]. The patient’s frequency of consultation with the GP prior to diagnosis can impact the stage of cancer diagnosis and therefore early diagnosis of cancer [26], especially since there is a strong association between GP-estimated cancer risk at referral and probability of cancer [27]. The GP, using a holistic approach, should be an attentive partner to share adequate decision-making in complex cancer treatments with the patient, in collaboration with other specialists [28]. Several qualitative studies have been published on patients’ perceptions of their cancer management [29], their relationship with the healthcare team [30] and their interest in medical education [31, 32]. Other studies have examined the perceptions of healthcare teams on their patients’ treatment [33–35]. These studies revealed a number of factors leading to communication difficulties between the patient, the oncologist and the GP. These difficulties can include a lack of direct communication between oncologists or GPs, the lack of two-way communication tools between professionals, the need to set up a patient centred management system and to allow a prolonged follow-up of patients by the GP. Furthermore the quality of the relationship between physician and patient could be a survival factor [36]. In our study, we found that increasing communication between different practitioners, in a way that is perceptible to the patient, reduces the use of emergency departments and increases the use of GPs in primary care. This makes it clear that by improving communication and coordination between GPs and oncologists, we have a direct impact on quality of life (less hospitalization, patient-friendly management) and on the patient’s survival [30–36].

Participation of the GP in diagnosis of cancer was estimated at 52.8% by patients. Three-quarters of patients had been followed by the same GP for more than 5 years, and half had been diagnosed within the previous year. There is a strong perception by the patient of the involvement of the GP in the diagnosis, as the GP follows the patient over the long term. The literature indicates that the investigations necessary for diagnosis are mainly prescribed by the GP [37]. The patient is then referred to the oncologist by the GP (passing from primary to hospital care) and once the pathology results are available the oncologist discloses the diagnosis of cancer. In France, the National Cancer Plan provides for a diagnosis disclosure procedure which consists of four consecutive phases: a medical phase (discussion between oncologist and patient), a phase of consultation on nursing and care provision needs, and a phase dealing with access to supportive care, while the last phase deals with communication with the GP [9]. During this last phase, the GP is informed about the diagnosis, the cancer treatment program, its side effects and the planned hospital consultations. In practice, this stage is often neglected; the literature finds that the GP is delegated to disclose the diagnosis to the patient in 19% of cases and that it is the patient who discloses their own diagnosis to the GP in 18% of cases [9]. Involving the GP in all the stages leading up to treatment optimizes follow-up and care coordination [38, 39]. Furthermore, the implication of the general practitioner in the health care decreases the number of visits to the emergency department [39, 40]. Our findings suggest that oncologists should inform the patient about the role of their GP. It thus seems indispensable for oncology departments to develop guidelines [41] to optimize patient-centered management, adapted to outpatient medicine. If the oncology department proposed that the patient should have a consultation with their GP following the diagnosis disclosure consultation, the GP would regain their role. The patient would then be in formal contact with their GP for management of their cancer, and a link would be created between the hospital and primary care. Jiwa et al. showed that this professional network between GP and other specialists impacts early diagnosis [42].

Our work underlines the need for communication between all those involved [20, 43], as well as the fact that this communication must be visible and made explicit to the patient. It probably increases confidence because we showed that when the patient believes that there is good communication between the GP and the oncologist, the number of patients consulting specifically for a cancer-related problem increases, whether in an emergency or not. The literature shows that GPs believe that there is room for improvement in their communication with oncologists [23, 44]. Although deeply involved in the management of patients with cancer, GPs feel isolated at crucial moments of the illness, and when making decisions at these times [33]. Many studies [12, 13, 17, 20, 44] reported unsatisfactory communication between GPs and oncologists. According to these authors, gaps exist in the transmission of information on patients to GPs. GPs felt that their specialist colleagues regarded them almost with contempt and, finally, that the specialists ‘captured’ certain of their patients. A qualitative study showed that GPs are more involved in the management of their patients with cancers because they have better access to communication with oncologists [45]. Another study [46] confirmed their desire to be involved in patient management and their regret regarding the poor communication with their oncologist colleagues [47]. Finally, patients themselves call for better cooperation between the physicians involved in their care [48]. Good communication between the different physicians is indispensable for good holistic patient care, particularly in complex situations [23, 24] or at the end of life [11]. Coordinating care for the patient suffering from cancer is affected by the relationship between the GP and the patient [23, 49].

### Implications

Systematically integrating a GP consultation in the cancer course of a patient could improve management and care coordination. Ideally, this involvement should be from the beginning, or after the announcement. This concept must be tested and evaluated in real practice, as a prospective study.

It could also be interesting to conduct co-training with oncologists and GPs. This would make it possible to develop a common care pathway between practitioners, for which communication would be planned and organised, allowing better coordination of care. Practitioners could use a common basis of advice and recommendation for supportive care and follow-up. The tools thus created could be used on electronic support for patient-centered coordination.

## Conclusion

The way in which patients perceive the relationship between their GP and their oncologist affects their healthcare utilization during their cancer care. The way in which the oncologist presents the GP’s role conditions patient management, and determines whether it will be possible in a primary care setting or not. This presentation is one of the keys of coordination between primary care and the hospital.

The general practitioner is an important contact and source of support for the patient. The GP must form an integral part of the medical team that will follow the patient throughout the management of the disease. Communication between the different physicians caring for the patient, together with communication between these physicians and the patient, is crucial for optimal management where each can fully play their own role. Further studies should demonstrate the impact of communication between the hospital and general practice in the management of patients suffering from cancer.

## Supplementary information

**Additional file 1.** Patients’ perspective on the role of their general practitioner in cancer management – French version. Questionnaire.

**Additional file 2.** Patients’ perspective on the role of their general practitioner in cancer management – English version. Questionnaire.

## Data Availability

The datasets used and analysed during the current study are available from the corresponding author on reasonable request.

## Abbreviations

GP: General Practitioner
RR: Risk Ratio
