# Supplementary material for: Improving communication between the general practitioner and the oncologist: a key role in coordinating care for patients suffering from cancer
Source: BMC Cancer. 2020 Jun 1;20:495. doi: 10.1186/s12885-020-06993-0 (PMC7268533; doi:10.1186/s12885-020-06993-0)
Supplement: Supplementary file 1 — Additional file 1. Patients’ perspective on the role of their general practitioner in cancer management – French version. Questionnaire. [file 12885_2020_6993_MOESM1_ESM.pdf]

UNIVERSITÉ TOULOUSE III PAUL SABATIER — Hôpitaux de Toulouse — INSTITUT UNIVERSITAIRE DU CANCER DE TOULOUSE Oncopole

"Point de vue des patients sur la place de leur médecin généraliste dans la prise en charge en oncologie"

Dans le cadre de mon mémoire de fin d'étude de médecine, je m'intéresse à votre point de vue sur la place que vous donnez à votre médecin généraliste dans le cadre de votre prise en charge actuelle.

Vous êtes.. un homme ☐ une femme ☐

Qu'elle est votre âge ?: . . . . . . ans

Vous et votre médecin généraliste

o Avez-vous un médecin généraliste ?...OUI ☐ NON ☐

o Depuis combien de temps ? (années)... 0-5 ☐ 5-15☐ 15 et +☐

o Avez-vous changé de médecin généraliste depuis le début de la prise en charge de votre maladie ?.. OUI ☐ NON ☐

o Quand avez vous vu votre médecin généraliste pour la dernière fois :

- -moins d'un mois...OUI ☐ NON ☐
- -entre 1 et 3 mois...OUI ☐ NON ☐
- -entre 3 et 6 mois...OUI ☐ NON ☐
- -6 mois ou plus...OUI ☐ NON ☐

Votre maladie et votre médecin généraliste

o Depuis combien de temps le diagnostic initial de cancer a-t-il été posé ?...0-1 an ☐ 1 an et plus☐

o Votre médecin généraliste a-t-il participé au diagnostic.................................... OUI ☐ NON ☐

o La dernière fois que vous avez vu votre médecin généraliste, était ce en lien avec votre maladie ?.. OUI ☐ NON ☐

o Pensez vous que votre médecin généraliste ait des connaissances sur votre maladie ?..OUI ☐ NON ☐

o Pensez vous qu'il reçoive régulièrement des informations concernant votre prise en charge à l'Oncopole ?..OUI ☐ NON ☐

o Pensez vous qu'il soit au courant du dernier changement de traitement ?...OUI ☐ NON ☐ Pas de changement ☐

Dans quelles situations consultez-vous votre médecin généraliste ?

o Consultez-vous votre médecin généraliste pour :

-vous conseiller sur les différentes possibilités de traitement de votre maladie ?...OUI ☐ NON ☐

-les effets indésirables du traitement ?..OUI ☐ NON ☐

- les éventuelles complications de la maladie ?..OUI ☐ NON ☐

-pour soulager la douleur ?...OUI ☐ NON ☐

Lors de l'urgence

o En cas d'urgence vous vous tournerez en priorité vers :

-votre médecin généraliste...OUI ☐ NON ☐

-les urgences...OUI ☐ NON ☐

-l'Oncopole...OUI ☐ NON ☐

o Pensez vous que votre médecin généraliste serait disponible en cas d'urgence..OUI ☐ NON ☐

o En cas d'urgence, pensez vous que votre médecin généraliste pourrait vous prendre en charge..OUI ☐ NON ☐

Votre médecin généraliste et votre oncologue

o Estimez vous que la communication entre votre oncologue et votre médecin généraliste est satisfaisante...OUI ☐ NON ☐

o Estimez vous que l'avis de votre médecin généraliste ait déjà été pris en compte dans les décisions médicales vous concernant...OUI ☐ NON ☐

o Votre oncologue a-t-il évoqué le rôle de votre médecin généraliste dans votre prise en charge..OUI ☐ NON ☐

Quel rôle donnez-vous à votre médecin généraliste au sujet de votre maladie ?

....

....

....

....

Merci beaucoup d'avoir participé à ce questionnaire, Vladimir Druel,

Interne des hôpitaux de Toulouse
