# Supplementary material for: Improving communication between the general practitioner and the oncologist: a key role in coordinating care for patients suffering from cancer
Source: BMC Cancer. 2020 Jun 1;20:495. doi: 10.1186/s12885-020-06993-0 (PMC7268533; doi:10.1186/s12885-020-06993-0)
Supplement: Supplementary file 2 — Additional file 2. Patients’ perspective on the role of their general practitioner in cancer management – English version. Questionnaire. [file 12885_2020_6993_MOESM2_ESM.pdf]

UNIVERSITÉ TOULOUSE III PAUL SABATIER — Hôpitaux de Toulouse — INSTITUT UNIVERSITAIRE DU CANCER DE TOULOUSE Oncopole

" Patients' perspective on the role of their general practitioner in cancer management "

You are.. a man ☐ a woman ☐

What is your age? ………… years

You and your general practitioner

- Do you have a general practitioner? .... YES ☐ NO ☐
- How long have you been with this doctor? (years) .... 0-5 ☐ 5-15 ☐ 15 and + ☐
- Have you changed your general practitioner since starting treatment for your disease? .... YES ☐ NO ☐
- When did you last visit your general practitioner:
  - Less than a month ago .... YES ☐ NO ☐
  - 1 to 3 months ago .... YES ☐ NO ☐
  - 4 to 6 months ago .... YES ☐ NO ☐
  - 6 months ago or more .... YES ☐ NO ☐

Your illness and your general practitioner

- How long ago was the cancer first diagnosed? ....0-1 year ☐ 1 year or more ☐
- Did your general practitioner take part in the diagnosis? .... YES ☐ NO ☐
- The last time you visited your general practitioner, was the visit related to your disease? .... YES ☐ NO ☐
- Do you think that your general practitioner has good knowledge about your disease? .... YES ☐ NO ☐
- Do you think that he/she regularly receives information about your treatment at the specialized cancer center? .... YES ☐ NO ☐
- Do you think that he/she knows about the most recent change in your treatment.. YES ☐ NO ☐ No change in treatment ☐

In what circumstances do you consult your general practitioner?

○ Do you consult your general practitioner about:

- advice on the treatment possibilities for your disease? .... YES ☐ NO ☐

- adverse effects of treatment? .... YES ☐ NO ☐

- complications, if any, of the disease? .... YES ☐ NO ☐

- pain relief? .... YES ☐ NO ☐

In an emergency

○ In an emergency, would you first turn to:

- your general practitioner... YES ☐ NO ☐

- emergency department .... YES ☐ NO ☐

- specialized cancer center .... YES ☐ NO ☐

○ Do you think that your general practitioner would be available in an emergency? .... YES ☐ NO ☐

○ In an emergency, do you think that your general practitioner can care for you? ...... YES ☐ NO ☐

Your general practitioner and your oncologist

○ Do you think that communication between your oncologist and your general practitioner is satisfactory? .... YES ☐ NO ☐

○ Do you think that your general practitioner's opinion has already been taken into account in medical decisions about your case? .... YES ☐ NO ☐

○ Has your oncologist talked about your general practitioner's role in your care ? .... YES ☐ NO ☐

What do you think is your general practitioner's role in relation to your disease?

....

....

....

....

Thank you for taking the time to complete this questionnairequestionnaire, Vladimir Druel,

Interne des hôpitaux de Toulouse
